# Magnetic resonance imaging does not reveal structural alterations in the brain of grapheme-color synesthetes

**DOI:** 10.1371/journal.pone.0194422

**Published:** 2018-04-04

**Authors:** Michel Dojat, Fabrizio Pizzagalli, Jean-Michel Hupé

**Affiliations:** 1 Grenoble Institut des Neurosciences, Université Grenoble Alpes, Institut National de la Santé et de la Recherche Médicale & Centre Hospitalier Universitaire Grenoble Alpes, Grenoble, France; 2 Centre de Recherche Cerveau et Cognition, Université de Toulouse Paul Sabatier & Centre National de la Recherche Scientifique, Toulouse, France; Centre de neuroscience cognitive, FRANCE

## Abstract

Several publications have reported structural changes in the brain of synesthetes compared to controls, either local differences or differences in connectivity. In the present study, we pursued this quest for structural brain differences that might support the subjective experience of synesthesia. In particular, for the first time in this field, we investigated brain folding in comparing 45 sulcal shapes in each hemisphere of control and grapheme-color synesthete populations. To overcome flaws relative to data interpretation based only on p-values, common in the synesthesia literature, we report confidence intervals of effect sizes. Moreover, our statistical maps are displayed without introducing the classical, but misleading, p-value level threshold. We adopt such a methodological procedure to facilitate appropriate data interpretation and promote the “New Statistics” approach. Based on structural or diffusion magnetic resonance imaging data, we did not find any strong cerebral anomaly, in sulci, tissue volume, tissue density or fiber organization that could support synesthetic color experience. Finally, by sharing our complete datasets, we strongly support the multi-center construction of a sufficient large dataset repository for detecting, if any, subtle brain differences that may help understanding how a subjective experience, such as synesthesia, is mentally constructed.

## Introduction

Various synesthetic experiences are reported by a potentially underestimated fraction, possibly up to about 20%, of the healthy population [1]. Synesthetes experience additional, systematic, arbitrary and involuntary associations. For instance, they may associate a specific color to some “graphemes” (the visual form of numbers or letters). To understand this subjective experience, we may hypothesize that some structural differences exist in the brain of synesthetes compared to controls that trigger and support these associations. In particular, the cross-activation theory considers increased connectivity between proximal regions; for grapheme-color association between areas involved in color perception and areas involved in grapheme recognition [2]. Several studies have searched for such extra-numerous connections using structural Magnetic Resonance (MR) images or Diffusion Tensor Imaging (DTI) (see [3, 4] for reviews). Indeed, structural imaging coupled with Voxel Based Morphometry (VBM) method [5, 6] appears as a powerful tool for detecting possible differences in the brain of synesthetes compared to controls. VBM assesses local (voxel-by-voxel) differences between populations of interest in tissue volume, mainly grey matter (GM) and white matter (WM) [7–10]. With DTI one can measure fractional anisotropy (FA) and then track again voxel-by-voxel possible differences of structural connectivity in the brain of synesthetes [11–15]. However, despites many reports of those, no structural differences might exist in the brain of synesthetes. Indeed, the careful examination of the reported differences concerning 19 studies on morphometry (n = 11) and structural connectivity (n = 8), indicates that no clear view emerges from the literature and suggests that the observed differences are false positives due to methodological issues [3].

The present paper pursues this quest for structural differences in the brain of synesthetes. First, we replicated our previously published study [8] on a new, larger, population of grapheme-color synesthetes. We obtained different results from those previously reported 1) in re-analyzing our initial data with an updated version of the initial VBM pipeline used, 2) in analyzing the new synesthete and control populations data and 3) in pooling the two sets of data. Second, to complement this morphometric analysis, we 4) searched for possible structural connectivity differences based on Mean Diffusivity (MD) and FA extracted from DTI data. Finally, and for the first time on a population of synesthetes, we 5) explored the possible anatomical differences at the level of the sulci architecture using a sulcal-based morphometry approach. In order to prevent from the severe flaws of null-hypothesis significance testing (NSHT) [16] and the difficult control of false positives [17], we adopted in this paper, the “New Statistic” approach [18] reporting confidence intervals (CIs) of effect sizes. Moreover, statistical maps were displayed without introducing the classical p-value level threshold, limiting data interpretation bias [19]. None of our analyses allowed us differencing reliably synesthete brains from control brains, based on either standard tissue volume, tissue density or fiber organization criteria, or newly introduced brain sulci descriptors. Following these analyses investigating different brain features, we conclude that a larger sample size for appropriate statistical power is mandatory to reveal if any, small, structural differences exist in the brain of synesthetes compared to controls. Multi-center data sharing is the realistic means to increase the synesthete population size to study. To launch such a data sharing process, we render our data publicly available (see Discussion Section).

## Materials and methods

### Data

We conducted two studies involving synesthetes, one during the period from 2010 to 2011, Study 1, described in [8] and a more recent study, Study 2. These studies were performed following project approval by the Institutional Review Board of Grenoble (CPP 12-CHUG-17) and written consent from the subjects. All subjects, but one, had normal color perception on the Lanthony D-15 desaturated color test (Richmond products). Synesthetic associations were strictly controlled and consistency checked using a modified version of the Synaesthesia Battery test [20].

#### Study 1

Ten grapheme-color synesthetes and twenty-five controls participated in this study. For each subject, we acquired structural images on a Bruker 3T Medspec S300 whole body scanner, equipped with a one channel emission/reception head coil, using a T1-weighted 3D MP-RAGE image consisting in 176 sagittal partitions in two segments with an image matrix of 256x112 (read x phase). Further imaging sequence parameters were: TR/TE/TI: 16/4.96/903 ms, excitation pulse angle: 8°, acquisition matrix: 176x224x256 (x,y,z), fast phase encoding in anterio-posterior direction (112 steps per RAGE train, 2 segments), slow phase encoding in left-right direction, isotropic nominal resolution: 1 mm, BW = 130 Hz/Px, readout in caudo-cranial direction, number of averages: 1 and total measurement time: 14 min 40 s.

#### Study 2

Twenty-two grapheme-color synesthetes and twenty-five controls participated in this study (a different volunteers’ population from Study 1). For each subject, we acquired a high-resolution structural MP-RAGE image on a 3T Philips Intera Achieva, using a 32 channels coil, 180 sagittal slices of 256x240 (read x phase). Further imaging sequence parameters were: TR/TE/TI: 25/3.7/800 ms, excitation pulse angle: 15°, isotropic nominal resolution: 1 mm, BW = 191 Hz/Px, readout in anterio-posterior direction, number of averages: 1, sense factor anterio-posterior: 2.2, right-left: 2 and total measurement time: 9 min 41 s. We also acquired diffusion-weighted images (DTI) using a DWI sequence (120x120 matrix size with 70 contiguous transverse slices, FOV = 240 mm, 2x2x1.75 mm^3^ spatial resolution, TR = 6845 ms, TE = 667 ms, FA = 90°, Sense Factor = 2 to improve the signal to noise ratio, total acquisition time: 18 min). The encoding protocol included 60 different non-collinear directions (gradient factor b = 1000 s/mm^2^), and one image without diffusion weighting used as reference volume.

Useful characteristics about the synesthetes and controls populations for the two studies are provided in S1–S3 Tables.

### Data processing of structural images

#### Voxel Based Morphometry (VBM)

We analyzed the structural images using the VBM approach [5] where region-wise volumetric comparison among groups of subjects is performed. It requires all individual images to be registered in the same space and segmented in different tissue classes. Then, registration parameters and a mixture of Gaussian distributions for brain tissue modeling have to be estimated. A generative approach has been proposed for such a model parameter estimation that alternates among classification, bias correction and registration [21]. We used the data processing pipeline VBM8 (http://www.neuro.uni-jena.de/vbm/) that implements this approach as a toolbox extension of SPM8 (http://www.fil.ion.ucl.ac.uk/spm/software/spm8/) running with Matlab language. Compared to the previous one, this new version mainly improves the segmentation step in removing noise [22], estimating partial volume effects [23], taking into account local intensity variations and ensuring local coherency of tissue labels [24]. A joint segmentation-registration approach is used to segment and warp the individual tissue probability maps into a common study-specific reference space. Then an affine registration may be applied for transformation into the Montreal Neurological Institute (MNI) referential space. Each structural image is segmented by attributing to each voxel a probability of being in white matter (WM), grey matter (GM), and cerebrospinal fluid (CSF). This procedure uses a maximum *a posteriori* estimation to take into account local variations of intensity and estimates a mixture model composed of several Gaussian distributions notably for pure tissue (3 gaussians) and partial volume effects (2 gaussians). To impose local coherence, a Markovian approach introduces spatial prior into the model estimation. The high-dimensional DARTEL [21] registration algorithm iteratively computes deformation fields for warping each individual image to the common space. To counterbalance local deformations, expansion, or contraction, induced by highly non-linear registration and affine transformation, the tissues’ probability values computed were scaled by the Jacobian determinants of the non-linear deformations (‘‘modulation step” [25]). Finally, following the recommendations of [26] for decreasing the false positive rate, we smoothed these ‘‘modulated” tissues probability maps using a 12-mm full-width at half-maximum Gaussian kernel (same pattern of results with an 8-mm kernel). A visual control of the sample homogeneity as implemented in VBM8 was realized based on the covariance between the images. No outlier was detected.

#### Diffusion Tensor Imaging

DTI images were first denoised [27] and preprocessed using FSL software (http://www.fmrib.ox.ac.uk/fsl/). The images were corrected for geometric distortions caused by Eddy currents and head movements (FSL “eddy” tool). The diffusion tensor was estimated, and the local diffusion FA and mean diffusivity (MD) parameters were calculated for the entire brain in each participant. These parameters were computed from the three estimated eigenvalues describing the water diffusion in three orthogonal directions. FA represents the coefficient of variation of these eigenvalues interpreted as the directionality of water diffusivity into fibers (coherence). MD is the mean of the eigenvalues and blurs the directionality. In general FA is noisier than MD. FA was considered for WM tracks only, MD for the entire brain. These parameters are myelination markers, serving as measures of tissue density for MD and fiber organization for FA.

For each participant, the non-diffusion weighted image (T2-weighted) was realigned to the corresponding structural image. The computed realignment parameters were applied to the corresponding MD volumes to be aligned to the structural image. Then they were warped using the deformation field previously computed for this image for warping to the common space, scaled by the Jacobian determinants of the deformations and smoothed (12 mm FWHM). Subsequently, MD volumes were analyzed on a voxel-by-voxel basis similarly to VBM-GM/WM volumes analysis [28]. Because such a voxel-based analysis does not ensure a proper alignment of individual fiber tracts, a Track-Based Spatial Statistics (TBSS) was also processed [29]. Individual FA data were nonlinearly realigned and a mean FA image was computed and used to define a mean FA skeleton on which all individuals FA data were projected. No spatial smoothing was applied to FA maps.

#### Sulci extraction and morphometry

“Sulcus-based morphometry” provides measures of the cortical fissures of the brain, which have been found to be associated with brain maturation [30, 31], brain alteration with age or pathology [32, 33], cognitive abilities [34–36] or correlated in a population of twins [37]. Because grapheme-color synesthesia seems to take place during the learning of reading [38] it was of interest to search whether some sulcal anatomical differences could be the cortical support of synesthesia experiences. We investigated sulci morphometry in controls and synesthetes based on shape descriptors (width, length, mean depth, and total surface area) analyzed for each sulcus. We used Freesurfer (https://surfer.nmr.mgh.harvard.edu) to classify grey and white matter tissues and Morphologist 2013, an image-processing pipeline included in BrainVISA (http://brainvisa.info/web/index.html), to quantify the sulcal descriptors. Briefly, the Morphologist 2013 segmentation pipeline computes a brain mask, imports brain tissues (gray, white matter and CSF) classified by Freesurfer, performs gray/white surface identification and spherical triangulation of the external cortical surface of both hemispheres. Sulci were then automatically labeled according to a predefined anatomical nomenclature [39]. The number of voxels on the junction between the sulcal mesh and the brain hull gave a measure of sulcal length. The mean depth was defined as the mean of geodesic distances computed for all voxel belonging to the sulcal fundus, from the bottom of the sulcus to the brain hull, along the cortical mesh. The surface area was the total area of the sulcal mesh. The sulcal width was obtained by dividing the enclosed CSF volume by the sulcal surface area; see [40] for details.

The pipeline failed for two control subjects, leaving 32 synesthetes and 48/50 controls. In addition, each sulcus could not be measured in every subject. We considered that a sulcus could not be measured for a given subject if the four measures (length, width, surface and depth) were equal to zero (that was the case for most zero values). Those cases appeared when individual variability was too high compared to the reference population. The pattern recognition algorithm then failed to sulcus identification, meaning that the corresponding sulcus was absent for this subject or not measurable. When a sulcus was not measured for more than 11% of subjects either in the control group or in the synesthete group (that was, in either 5/48 subjects in the control group or 3/32 subjects in the synesthete group), it was removed from the analysis for the two hemispheres (it does not make sense to compare different sulci if tested on too many different subjects). When a sulcus was missing for a subject, we attributed the median value of all other subjects (controls and synesthetes, left and right sides, independently) for that sulcus. The interpolated value was used only for the sum and the subsequent normalization. We normalized each measure by dividing the within subject sum of the sulci independently for length, depth, surface and width. We computed Pearson’s correlation between left and right sulci values to consider pooling the right and left values or not.

### Statistical analysis

#### Structural images

We compared the regional tissue probability maps (modulated and smoothed as described above) of controls and synesthetes by performing a voxel-wise univariate analysis using the general linear model (GLM) as implemented in SPM8. Because the global brain size can vary across subjects, we included brain volume as a factor of noninterest in our statistical tests. In order to calculate the global brain volume, we used the modulated images by summing together the GM and WM probabilities of all voxels. To avoid possible edge effects between different tissue types, we applied an absolute intensity threshold mask of 0.1 on each tissue probability.

For Study 1, average age slightly differed between the 2 groups (29.8 *vs*. 36.4 years, p = 0.13), and our synesthete group had more women (7/10 *vs*. 10/25 in our control group). Both factors may generate local differences not related to synesthesia, so we also included sex and age as factors of noninterest. In Study 2, the two groups were not different in term of age (28.2 *vs*. 27.7 years) and sex (18/22 *vs*. 18/25 women). However, in order to compare the two studies, we adopted the same model including sex and age as cofactors at the expense of the number of degrees of freedom.

#### Diffusion Tensor Imaging

For MD data, we performed (Study 2) a voxel-wise univariate analysis using a GLM model similar to that used for tissue analysis. We considered MD, by definition an average value in each voxel, as a MR biomarker in the same way as GM or WM measures [41, 42].

There are several pitfalls using VBM to analyze DTI data mainly due to alignment issues. The use of the ‘FA skeleton’ and the projection of FA values onto it is an interesting approach that improves individual images alignment; moreover, it reduces the need for smoothing and the number of voxels to be tested; so, we used this TBSS approach as implemented in FSL to compare FA projections onto the mean population skeleton between synesthetes and controls. Note that in this case the analysis is reduced to white matter only.

We used two different statistical approaches, the Random field Theory (RFT) for MD and voxel-wise permutation tests [43] for FA, in order to have the best method adapted to the measures we have. Moreover, using the statistical tools commonly used for FA and MD allows the comparison of our results with the literature. The RFT cannot be applied to the tracks of the skeleton (a track is definitely not a random field). Permutation tests are valid because they deal only with group exchangeability; unfortunately, permutation tests do not allow any meaningful inference since they do not allow building up confidence intervals of effect sizes, because they cannot test any other hypothesis than the null hypothesis [44]. These tests allow only the computation of p-values. The only conclusion based on a permutation test when getting a small p-value is that the observed sample is surprising if we suppose exchangeability (so the two groups may not be exchangeable). This does not provide much information but may yet constitute a starting point: finding locations with small p-values indicates regions where it is worth exploring the data in more details. In order to obtain at least p-values as accurate as possible for the FA measure, we performed cluster-based statistics with the "threshold-free cluster enhancement" (TFCE) approach as implemented in FSL “randomize” function [45].

#### Sulci

We compared the distribution of sulcal length, mean depth, surface and width between the two populations across both studies for right and left hemispheres. We computed 99.9% CI for the group difference using a linear model, with age and sex as covariates. We chose quite arbitrarily the 99.9% value to partially account for multiple comparisons (4 measures in 61 sulci in each hemisphere) while avoiding being too conservative, because of possible correlations between measures (a 99.9% CI corresponds to a 95% family-wise CI for 50 independent comparisons). We could not perform multivariate analyses because of the large number of missing values (not all sulci could be identified in every subject).

#### Data exploration

In order to compare the results of Study 2 with our results published in 2012, we considered as a starting point the family wise error (FWE correction for multiple comparison) measured at the cluster level. We defined two regions of interest (spheres, radius 6 mm) located at the coordinates of the clusters found in [8] that survived a strict FWE correction, in the right retrosplenial cortex (RSC) and the left superior temporal sulcus (STS). Two additional regions in the fusiform gyrus and in the parietal cortex were considered. For each sphere, we computed in both studies WM estimated using our general linear model for the two groups. Recently Eklund et al. [17] showed that such a multiple comparison correction procedure did not guarantee at all the control of false positives at a cluster level. Besides standard p-values for comparison with published studies, we reported CIs of effect sizes to facilitate appropriate data interpretation in context. Moreover, our statistical maps are displayed without introducing the classical p-value level threshold. We adopt the dual-coding approach proposed in [19] where differences in effect size (beta estimates) are color-coded and associated t-statistics mapped to color transparency.

## Results

### Reproducibility tests

#### Study 1

**Fig 1:**
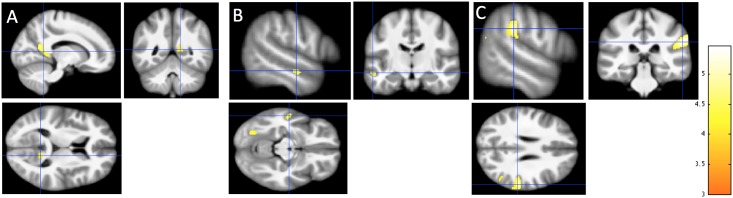
Local increases of WM in synesthetes compared with controls. Detected changes are projected onto the study-specific structural image transformed in the MNI space. (A) Increase in the right RSC (14, -57, 6). (B) Increase in the left anterior middle temporal gyrus (Blue cross: -56–15–14). Note the second cluster in the posterior part located in the fusiform gyrus (-26–73–12). (C) Increase in the right inferior parietal lobe (58–31 28). All coordinates are in MNI space expressed in mm. Cluster-forming threshold p<0.001 uncorrected, t>3.4, k>70 voxels i.e 70 mm^3^. T scale is between 3 and 5.5. Neurological convention (right hemisphere displayed on the right side of the figure).

We compared the local distributions of WM and GM in the brains of 10 synesthetes with the brains of 25 controls. In order to replicate our previous results [8] we first used the same, p-value based, statistical analysis conditions (cluster forming threshold at p<0.0001, cluster extent k>70) but we did not obtain any cluster for WM or GM. At a more lenient threshold (p<0.001, the default cluster-defining threshold in SPM) we found local increases of WM in synesthetes compared to controls in the right retrosplenial cortex (Fig 1A) and the left anterior middle temporal gyrus (Fig 1B), similarly to [8] (see their Fig 6). However, these clusters were found at a statistical threshold non-corrected for multiple comparisons (see Table 1). At this threshold new clusters appeared (see Table 1) in the right inferior parietal lobe (Fig 1C) and in the vicinity of color-sensitive regions of the fusiform gyrus [8, 46–48] (posterior cluster in Fig 1B). These two regions were close to some reported results [9, 10, 12].

**Table 1:** Local increase of WM in synesthetes (n = 10) compared with controls (n = 25).

	Cluster size (mm^3^)	x (mm)	y (mm)	z (mm)	Max t-value	FWEc
Right inferior parietal lobe	911	58	-31	28	5.43	0.141
Right RSC	489	14	-57	6	4.54	0.334
Left fusiform gyrus	187	-26	-73	-12	3.96	0.365
Left STS	86	-56	-15	-14	4.10	0.779

Note: (x, y, z) = MNI coordinates of the center of each cluster. Max t-value is the voxel maximum in the corresponding cluster. FWEc is the p-value corrected for the FWE at the cluster level. We obtained these 4 clusters when thresholding p<0.001 for individual voxels, with a minimum cluster size of 70 voxels i.e. 70 mm^3^.

**Fig 2:**
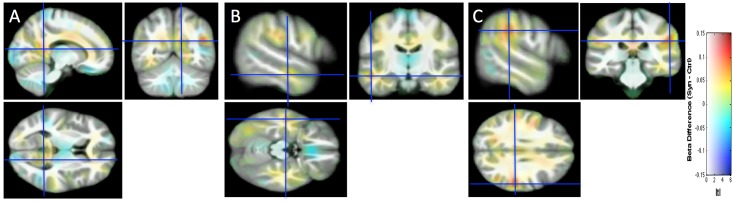
The structural differences between the two groups are projected without the introduction of a threshold onto the study-specific structural image transformed in the MNI space. Same slices, convention and cursor positions as in Fig 1. Beta difference scale is between -0.15 and 0.15 and mapped to color hue. t-statistic magnitude (| t |) is mapped to color transparency.

Fig 2 displays structural differences without the introduction of a threshold.

**Fig 3:**
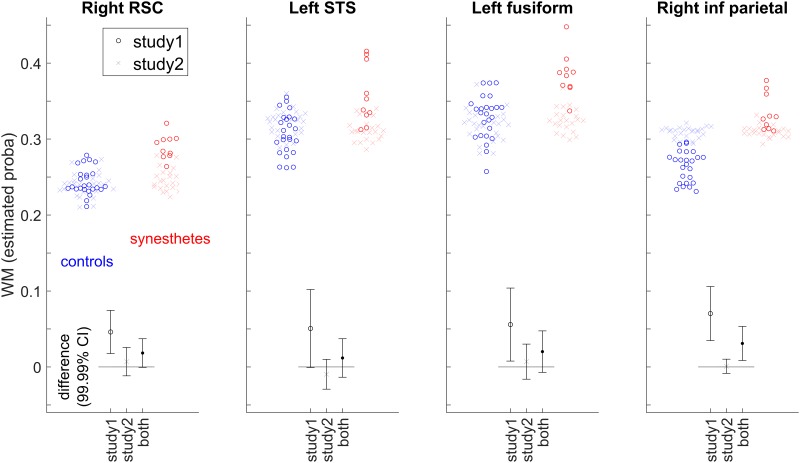
WM in synesthete and control groups, and 99.99% CI for the group difference, for four regions of interest centered in the right RSC, left STS, the right inferior parietal lobe and the left fusiform gyrus (coordinates in Table 1), for the subjects of Study 1, Study 2, and all subjects together. The WM tissue probability at each voxel was estimated by the linear model that included total brain volume, age and sex as covariates.

Fig 3 shows the individual data (denoted by circles for this first study) for the four regions of interest identified in Fig 2, using 6-mm spheres centered on the peak coordinates of Table 1, as well as the 99.99% CI (corresponding to the arbitrary voxel-wise threshold of our statistical map used to identify clusters). By construction, the extent of each CI of the group difference was expected to be above zero, since each value is the average of voxels with p-values < 0.001 for the group comparison. Note however that for small clusters (like in the left STS) this was not necessarily the case since the 6-mm sphere may extend beyond the cluster of small p-value voxels.

#### Study 2

**Fig 4:**
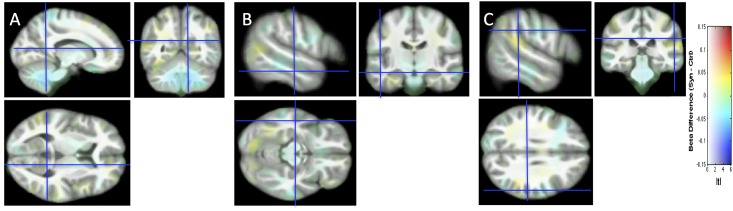
Study 2. Structural differences between the two groups are projected without the introduction of a threshold onto the study-specific structural image transformed in the MNI space. Same slices, convention and cursor position as in Fig 1.

We compared the local distributions of WM and GM in the brains of 22 synesthetes with the brains of 25 controls. Fig 4 shows the structural differences of WM without introducing any arbitrary threshold, in the same slices as for Study 1. Fig 4 (crosses) indicates that structural differences are unlikely (or too small to be relevant and detectable) in the regions of interest defined in the first study (see also the CI of group differences for Study 2 in Fig 3). Using conventional 5% family-wise error risk, we did not find any ground (increase or decrease) for rejecting the Null hypothesis of no difference between the two groups. We obtained the same result for GM analysis. Following the protocol proposed by Kurth et al [49] we searched to assess whether the voxel-wise gray matter asymmetry in the synesthete group was significantly different from the voxel-wise gray matter asymmetry in the control group. No difference was found using a standard p<0.001 for individual voxels.

#### Pooling data from Study 1 and Study 2

**Fig 5:**
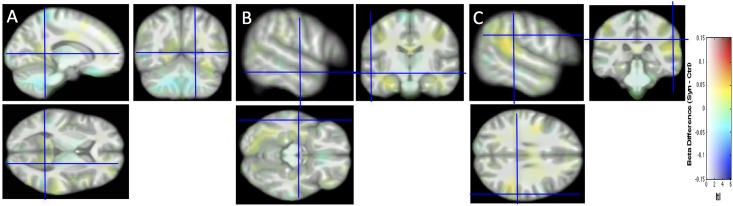
All the structural differences between the two groups are projected without the introduction of a threshold onto the study-specific structural image transformed in the MNI space. Same slices, convention and cursor position as in Fig 1.

Here we considered pooling data from Study 1 and Study 2 (synesthetes = 32, controls = 50). Because data were acquired on two different scanners, 3T Bruker for Study 1 and 3T Philips for Study 2, we were particularly meticulous with the quality check after the preprocessing including realignment and segmentation steps. We used the module available in VBM8 for checking homogeneity of the segmented volume GM and WM separately using covariance. We added in our general linear model a fourth covariable of non-interest corresponding to the two different conditions of data acquisition. Fig 5 shows all the WM differences without introducing any arbitrary threshold. Fig 3 shows the CIs for the regions considered in Figs 2 and 4. Two clusters emerged at our threshold, none of them allowing us to reject the null hypothesis at the 0.05 FWE level (see Table 2).

**Table 2:** Local increase of WM in synesthetes (n = 32) compared with nonsynesthetes (n = 50).

	Cluster size (mm^3^)	x (mm)	y (mm)	z (mm)	Max t-value	FWEc
Right superior temporal lobe	823	68	-37	18	4.13	0.186
Left fusiform gyrus	76	-44	-43	-29	3.94	0.301

Note: (**x**, **y**, **z**) = MNI coordinates of the center of each cluster. Max t-value is the voxel maximum in the corresponding cluster. FWEc is the p-value corrected for the FWE at the cluster level. We obtained these 2 clusters when thresholding p**<**0.001 for individual voxels, with a minimum cluster size of 70 voxels i.e 70 mm^3^.

In order to provide an idea of the power of these “Null” results, one may consider the upper (for increases) and lower (for decreases) limits of the CIs. For the increases in synesthetes relative to control reported in Fig 3, the largest differences of WM probability compatible with our measures in the 4 regions of interest were about 0.05 (based on a 99.99% CI). In the voxels with the maximal statistical differences reported in Fig 5 (max t-value = 4.39 and min t-value = -4.90) the upper and lower limits of the WM probability differences were respectively 0.0555 and -0.0525.

For 29/32 synesthetes, we had measured the strength of associations (“photism strength”), estimated during psychophysics Stroop-like tasks [50] (see S1 Table). We inserted this value as a covariate in our design matrix, in addition to brain volume, sex, age and scanner, and searched for structural data correlations using the VBM approach. The reasoning was that if effects, even weak, were found in the data, correlation with photism strength would have indicated a direct relationship with the synesthetic experience. Correlations were between -3x10^-4^ to +3x10^-4^, with no brain region exhibiting any reliable correlation (see S1 Fig). Results were similar for GM data.

### DTI

**Fig 6:**
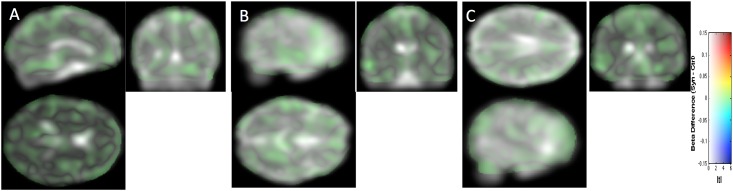
All the mean diffusivity differences between the two groups are projected without the introduction of a threshold onto the MD image of one subject transformed in the MNI space.

We compared the local distributions of MD in the brains of 22 synesthetes with the brains of 25 controls. We did not to find any “statistically significant” difference using the p-value based procedure as done for WM and GM tissue (cluster-forming threshold p<0.001 uncorrected, t>3.4). Fig 6 shows all the differences without the introduction of an arbitrary threshold at the same spatial coordinates we used for brain tissue comparison.

We also did not find any “statistically significant” difference of FA data, computed using permutation tests and TFCE cluster-based statistics, performed on 21 synesthetes (the nonlinear registration failed for one individual) versus 25 controls. An exploratory analysis (i.e. cluster-forming threshold p<0.05 uncorrected, without any correction for multiple comparisons at the cluster level; note that on average the detected tracks represented 9%—114599 voxels—of the brain volume), led to three clusters of at least 10 voxels of increase in FA, reported in Table 3, and decrease in FA, reported in Table 4, for synesthetes compared to controls.

**Table 3:** Local increase of FA in synesthetes (n = 21) compared with controls (n = 25).

	Cluster size (mm^3^)	x (mm)	y (mm)	z (mm)	Max t-value	FWEc
Right frontal cortex	101	26	27	5	4.74	uncorrected
Right caudate nucleus	26	8	-2	9	4.41	uncorrected
Right post central gyrus	10	26	-34	56	4.11	uncorrected

Note: (x, y, z) = MNI coordinates of the center of each cluster. Max t-value is the voxel maximum in the corresponding cluster. We obtained these 3 clusters with a non-parametric permutation testing analysis when thresholding p**<**0.05 for individual voxels uncorrected for multiple comparisons.

**Table 4:** Local decrease of FA in synesthetes (n = 21) compared with controls (n = 25).

	Cluster size (mm^3^)	x (mm)	y (mm)	z (mm)	Max t-value	FWEc
Middle frontal gyrus	16	32	35	1	3.86	uncorrected
Left superior frontal gyrus	14	-18	50	3	3.48	uncorrected
Right inferior frontal gyrus	14	34	35	5	3.8	uncorrected

Note: (**x**, **y**, **z**) = MNI coordinates of the center of each cluster. Max t-value is the voxel maximum in the corresponding cluster. We obtained these 2 clusters with a non-parametric permutation testing analysis when thresholding p**<**0.05 for individual voxels uncorrected for multiple comparisons.

**Fig 7:**
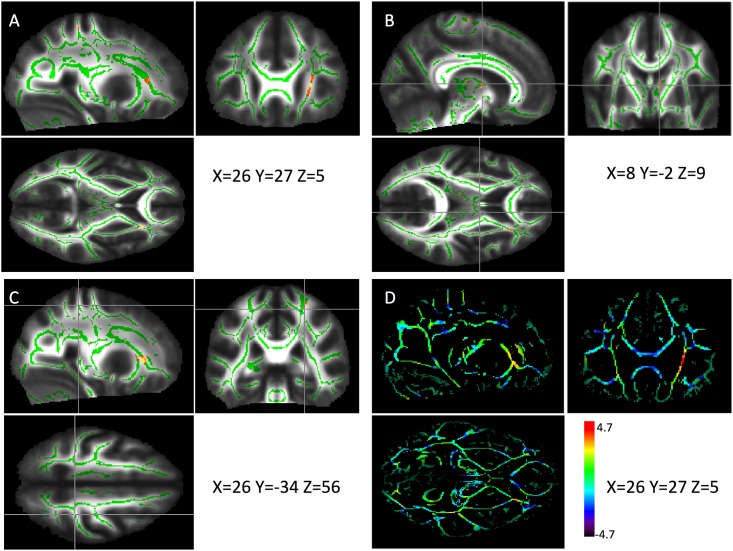
FA data. A, B, C: Increase in FA (in yellow-red) for synesthetes *vs*. controls (p<0.05) projected onto the mean FA image (white matter skeleton in green). D: All FA differences between the two groups without the introduction of a threshold.

Fig 7 shows the differences when applying a threshold at the voxel level (p<0.05, panels A to C) and without the introduction of an arbitrary threshold (panel D). No cluster survived the application of a correction for multiple comparisons (TFCE) at p<0.05.

**Fig 8:**
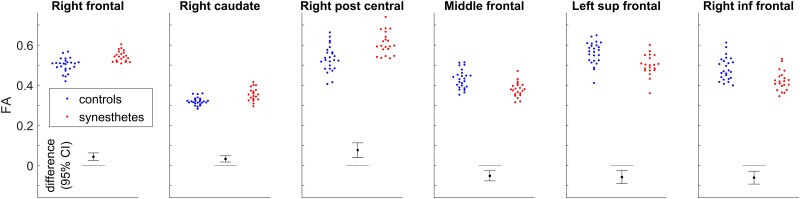
FA in synesthete and control groups, and 95% CI (no correction for multiple comparisons) for the group difference, for the six clusters identified in Tables 3 and 4 (same order); the first three graphs correspond to the increases represented in red in Fig 7.

Fig 8 shows the distributions of FA mean values in the clusters identified in Tables 3 and 4, in controls and synesthetes, as well as the 95% CI of the group difference (no correction for multiple comparisons, age and sex were included as covariates).

### Sulci morphometry

**Fig 9:**
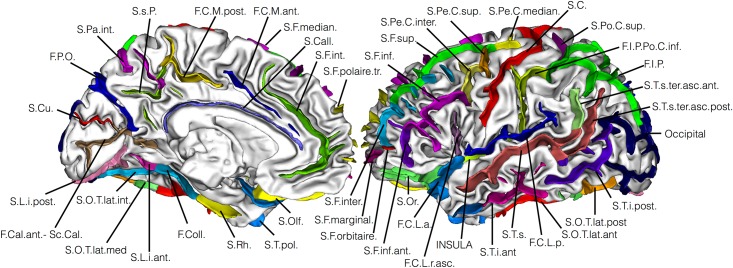
The 45 sulci we considered. See Table 5 for the abbreviations signification.

Sixty-two sulci for the left hemisphere and sixty-one sulci for the right hemisphere were extracted for each individual for Study 1 and Study 2 (for the list of extracted sulci based on the Brainvisa sulci atlas see [39]). Our criterion on missing values (see Materials and methods) allowed us to have only up to 5/48 controls or up to 3/32 synesthetes with at least a missing sulcus leading to the exclusion of 16 sulci on 61 in each hemisphere (see Fig 9). We normalized each value by dividing the within subject sum of the 90 sulci (45 right and 45 left), independently for length, depth, surface and width. Correlations (Pearson score) varied widely between corresponding left and right sulci, preventing from averaging the left and right values.

**Table 5:** Abbreviations used in Fig 9.

F.C.L.a.	Anterior lateral fissure
F.C.L.p.	Posterior lateral fissure
F.C.L.r.asc.	Ascending ramus of the lateral fissure
F.C.M.ant.	Calloso-marginal anterior fissure
F.C.M.post.	Calloso-marginal posterior fissure
F.Cal.ant.-Sc.Cal.	Calcarine fissure
F.Coll.	Collateral fissure
F.I.P.	Intraparietal sulcus
F.I.P.Po.C.inf.	Inferior postcentral intraparietal sulcus
F.P.O.	Parieto-occipital fissure
INSULA	Insula
OCCIPITAL	Occipital lobe
S.C.	Central sulcus
S.Call.	Subcallosal sulcus
S.Cu.	Cuneal sulcus
S.F.inf.	Inferior frontal sulcus
S.F.inf.ant.	Anterior inferior frontal sulcus
S.F.int.	Internal frontal sulcus
S.F.inter.	Intermediate frontal sulcus
S.F.marginal.	Marginal frontal sulcus
S.F.median.	Median frontal sulcus
S.F.orbitaire.	Orbital frontal sulcus
S.F.polaire.tr.	Polar frontal sulcus
S.F.sup.	Superior frontal sulcus
S.Li.ant.	Anterior intralingual sulcus
S.Li.post.	Posterior intra-lingual sulcus
S.O.T.lat.ant.	Anterior occipito-temporal lateral sulcus
S.O.T.lat.int.	Internal occipito-temporal lateral sulcus
S.O.T.lat.med.	Median occipito-temporal lateral sulcus
S.O.T.lat.post.	Posterior occipito-temporal lateral sulcus
S.Olf.	Olfactory sulcus
S.Or.	Orbital sulcus
S.Pa.int.	Internal parietal sulcus
S.Pe.C.inter.	Intermediate precentral sulcus
S.Pe.C.median.	Median precentral sulcus
S.Pe.C.sup.	Superior precentral sulcus
S.Po.C.sup.	Superior postcentral sulcus
S.Rh.	Rhinal sulcus
S.s.P.	Sub-parietal sulcus
S.T.i.ant.	Anterior inferior temporal sulcus
S.T.i.post.	Posterior inferior temporal sulcus
S.T.pol.	Polar temporal sulcus
S.T.s.	Superior temporal sulcus
S.T.s.ter.asc.ant.	Anterior terminal ascending branch of the sup. temp. sulcus
S.T.s.ter.asc.post.	Posterior terminal ascending branch of the sup. temp. sulcus

**Fig 10:**
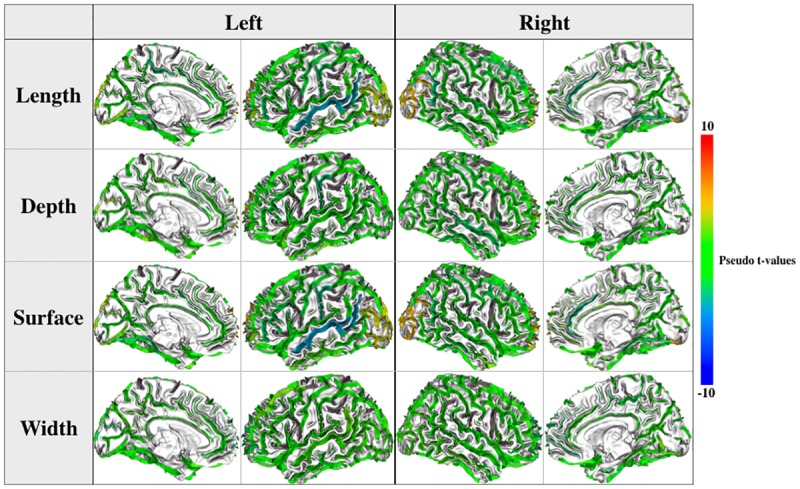
Pseudo t-values (square root of the F-score obtained in a linear model with age and sex as covariates) for the differences between the two groups for our four measures. Sulci with no values appear in grey.

Fig 10 shows t-scores for the differences between the two groups for our four measures. Similarly to Fig 5, we adopted a color scale that would highlight with salient colors (red and blue) only regions with potentially interesting differences, i.e. when the whole CI would be away from zero by at least one standard error above the mean (equivalent to t-values above 4.7; the t-threshold when correcting for multiple comparisons is about 3.7). With this representation most of the sulci, with greenish color, show no difference between the two groups. No region shows large differences (there is no dark blue or red regions). Finally, the occipital lobe, the superior temporal sulcus and the polar frontal sulcus show some differences both in the right and left hemisphere. Additionally, based on the cortical volume thickness and surface measures coming with Freesurfer (see Materials and methods) we performed a surface based analysis. No difference was found in cortical volume, thickness and surface measurements for each hemisphere for synesthetes *vs*. controls (cluster-forming threshold p<0.001). However, weak differences appeared for controls *vs*. synesthetes, but none in the left superior temporal sulcus (compare Fig 10 with S2 Fig).

**Fig 11:**
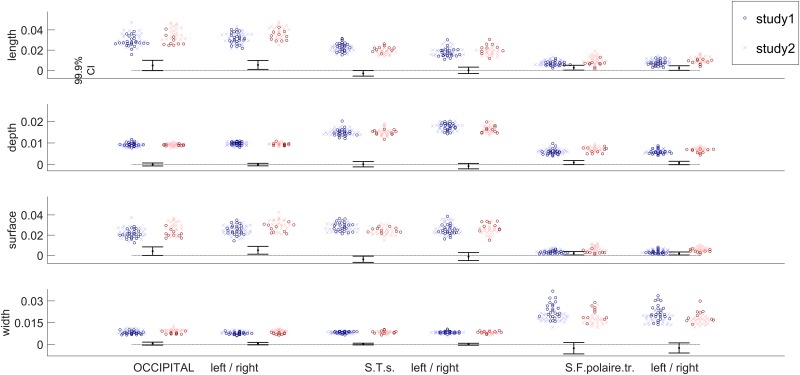
Sulcal length, mean depth, surface and width normalized for the total value in controls (blue) and synesthetes (red), in Study 1 (dark crosses) and Study 2 (bright dots) for three regions that show some differences. CIs were computed with age and sex as covariates (no interaction term).

In Fig 11 we report the individual values for the regions identified in Fig 10 as well as the 99.9% CI of the group difference (arbitrary correction for multiple comparisons; see Materials and methods). The results did not suggest any real difference between both groups.

We searched for correlations between photism strength and sulci measures. We computed the correlations for 90 sulci (45 on each side) and 4 measures. P-values for correlations were occasionally < 0.05 but the smallest p-value was 0.003, corresponding to a 5% family-wise correction over 19 tests only.

## Discussion

Several publications have reported structural brain alterations in synesthetes compared to controls. A recent review [3] shows a lack of consistency of the localization of the reported alterations and more importantly points out several methodological flaws. Our goal here was also to question the results we obtained in a recent study on graphemes-color synesthesia [8] and extend our study in introducing new features for anatomical comparison.

### Replication of a VBM study

Some image processing steps may influence the quality of voxel based morphometry results, especially spatial normalization and segmentation procedures [51–54]. Firstly, we reanalyzed our 3T Bruker data with an updated implementation of the VBM method (VBM8) and the same general linear model. The results show (see Table 1 and Fig 1) that the differences initially observed in the RSC and STS were detected but were weak (notably, they did not reach the classical “statistical threshold” anymore). Clearly, the improvement of the segmentation algorithm has an impact on the robustness of the detected differences. A small difference in the fusiform gyrus was revealed (X = -26, Y = -72, Z = -12). Several authors reported this region as color-sensitive. For instance, the color-sensitive area was defined in human at -27–57–11 [48]; -33, -65, -14 [46]; -29–68–14 [47] and -26–81–9 [8] (see [8] for a detailed discussion about the “color center” localization). Change in this region has been considered as the main cause for color synesthesia [55, 56]. However, recent attempts to find differences in this region were not convincing (see [3]). Our results (see Fig 2) revealed all structural differences including those not passing an arbitrary threshold and suggested that the screening of a larger synesthetes population could reveal significant differences.

For replication, we considered a new population of grapheme-color synesthetes (n = 22) and controls (n = 25), and analyzed the 3T Philips data with VBM8 using the model previously used. The comparison of Figs 2 and 4 indicates that potential structural differences were detected at different spatial locations based on either Study 1 or Study 2. When we pooled the two populations (32 *vs*. 50 subjects) some differences detected in Study 1 (see Fig 5, Table 2) in the fusiform gyrus were still visible. Two different MR scanners were used and because the ratio of cases to control was different between the studies, a covariate was introduced in our model for these two different acquisition conditions [57]. Pooling data from different scanners introduced confound in VBM analysis [58]. But because the value of the magnetic field (3T) and the spatial resolution of the structural images were identical, the “scanner effect” or “sequence effect” may be limited [59]. Note that if the initially detected differences were “real” (i.e. not due to sampling noise), they would be likely detected with a new MR scanner generation equipped with a 32 channels head coil (Study 2). The extent of the CIs for the group differences displayed in Fig 3 provides an idea of the order of magnitude of “true” differences compatible with our sample in Study 1, Study 2 and their pooling. There could be in fact no difference at all, but differences of up to 0.05 of WM probability are also compatible with our data. Such a difference (for example a change from 0.3 to 0.35) may be physiologically relevant, but could be assessed with confidence only by testing many more subjects.

### Structural connectivity

In order to search for other possible structural differences in the two populations, we acquired DTI data. Using MD (see Fig 6) we failed to detect differences. A local increase of FA would reveal more white matter and potentially more local connections (hyperconnectivity). Tables 3 and 4 indicate respectively local increase and decrease in FA in the brain of synesthetes. Fig 7 shows increase in FA for some clusters. The CI values for the group difference indicate that the measured differences were weak (Fig 8). Our results did not support previous claims using the same TBSS analysis procedure with [13, 14, 60] or without [15, 61] *a priori* hypothesis. Since the first attempt to detect structural differences in the brain of synesthetes using DTI [14], several improvements have been performed, from image acquisition (e.g. increase in the number of gradient directions), processing (e.g. geometric distortion correction) and analysis (e.g. crossing-fiber detection). A larger group study using state-of-the-art DTI acquisition and analysis procedures is needed for further structural connectivity investigation in synesthetes.

### Sulcal shapes

Finally, using a recent sulci-based morphometry technique [39] we searched for differences at the sulcal level. We considered four features: length, depth, width and surface for 45 sulci (see Fig 9). Fig 10 shows all the measured differences for these features for 32 synesthetes versus 48 controls. It clearly appears that no important difference in cortical anatomies was found between controls and synesthetes. Only weak differences appear in the superior temporal sulcus and the occipital lobe. The former result may be in line with the positive nonparametric correlation (r = 0.548, p = 0.009) between FA in the right temporal cortex and projector-associator scores (degree to which synesthetes experience the synesthetic color as projection or association) reported in [14]. However, with our score of photism strength [50] we did not find such a correlation. The difference in the occipital lobe is in coherence with our VBM results of Study 1 (see Fig 2). Fig 11 indicates the corresponding confidence intervals. Our study reports the first investigation of sulcal shapes in synesthetes compared to controls, but due to large individual variations of sulcal anatomy, processing a large cohort is required to assess the possible relationship between brain folding and synesthetic experiences. For instance [37] involved 1009 healthy young adults for understanding genetic factors contributing to sulcal shape variability.

### Shifting from NHST

To facilitate comparison with the literature we have reported p-value corrected for multiple comparisons. Eklund et al. [17] demonstrated that multiple comparisons correction procedures used in neuroimaging for cluster-wise inference artificially inflate false-positive rates. They considered that cluster failure for fMRI data inferences was mainly due to the false assumption of the Gaussian shape of spatial autocorrelation functions. Similarly for VBM studies, the difficulty to control false positive is due to the non-normality of the data, even after spatial smoothing, and directly dependent on sample size [62, 63]. More generally, Ioannidis [16] underlined that multiple testing corrections do not prevent from false positive findings, especially in life science where studies are in general underpowered face to the large set of potentially influent variables difficult to master. In the same line, several authors have emphasized that null hypothesis significance testing (NHST), leading to the unsolvable problem of false positive control, is a theoretical framework maladapted to determine the significance of the results in social, psychological and cognitive sciences and should be banished (see the virulent attack from the statistician J. Cohen [64] or [65]; see also [44] for MRI studies). A statistic paradigm shift is then proposed with the ‘New Statistics’ [18] reporting confidence intervals of effect sizes rather than p-values or, for neuroimaging studies, statistical maps at a predefined p-value threshold. Several arguments support moving beyond NHST towards a cumulative quantitative approach where data are presented in a way that facilitates their interpretation in context. We promote such an approach in our paper with the dual-coding data visualization proposed by [19] to complement CIs that can be computed only in regions of interest.

It is important to note that the samples of synesthetes tested in our two studies have no reason (to our knowledge) to be different from those involved in other published studies, for instance in the degree of synesthetic experience reported. In fact, published results are very comparable: never any larger differences than in our study have been reported, only over-emphasis of small and unreliable differences.

### Data sharing

As reported by several authors [66, 67], low statistical power is endemic in Neurosciences. Our study clearly demonstrates that the use of a small-size population leads to unreproducible results even when inclusion criteria for group definition are strict and false-positive rate “controlled”. Compared to other studies, our samples sizes were a bit larger than all but one [9], but still not enough to detect small effects. Data sharing between laboratories is the only way to improve statistical power in our neuroscience studies, increase reliability and confidence regarding effect size. This is more pregnant in domains, such as synesthesia, where the recruitment of subjects is costly, long and time-consuming and the effect to measure, if any, is subtle, as evidenced in the present study.

Knowledge about this subject will progress only if we decide to share our data. It is then imperative that all the steps of data processing be accurately reported and precise rules for statistical analysis respected (see [68, 69]for interesting recommendations). The ideal number of subjects for detecting a reproducible difference is not straightforward. Because the effect size may be low, more than one hundred individuals in each group seems a minimum [70]. For going in this direction, all our structural data (T1-weighted and DTI) are freely available on request (https://shanoir.irisa.fr/Shanoir/login.seam, contact M. Dojat). Please refer to the present paper in case of the reuse of these datasets.

## Supporting information

S1 Fig Correlation between WM and photism strength in our population of synesthetes. Left: Cluster-forming threshold p<0.001, t>3.4. Right: T statistic magnitude is mapped to color transparency. Correlation scale is between -2 and 2 10^−4^. For the coordinates for A, B and C views see the legend of Fig 2. Neurological convention (Right = Right).
(PDF)

S2 Fig Surface based morphometry. Differences in 48 controls *vs*. 32 synesthetes for cortical thickness (mm), area (mm^2^) and volume (mm^3^) for left and right hemispheres (Cluster-forming threshold p<0.001). For each parameter, regions where differences were detected are indicated. No region survived the FWEc at the cluster level.
(PDF)

S1 Text Surface based morphometry. Material and Methods.
(PDF)

S1 Table Individual demographic characteristics for the synesthete population. Average response time (inverse of the mean inverse RT) in synesthetic Stroop tasks: CC: Congruent stimuli in Color task; IC: Incongruent Color; CP: Congruent Photism task; IP: Incongruent Photism. “Synesthesia Strength” (also called photism strength) = (IC-CC)–(CP-CC). (see for details in [48]).
(PDF)

S2 Table Individual demographic characteristics for the control population. * subject not used in sulci and surface-based morphometry analyzes.
(PDF)

S3 Table Mean demographic characteristics for the control and synesthete populations. (PDF)
