# Supplementary material for: Magnetic resonance imaging does not reveal structural alterations in the brain of grapheme-color synesthetes
Source: PLoS One. 2018 Apr 4;13(4):e0194422. doi: 10.1371/journal.pone.0194422 (PMC5884511; doi:10.1371/journal.pone.0194422)
Supplement: S1 Fig — Left: Cluster-forming threshold p<0.001, t>3.4. Right: T statistic magnitude is mapped to color transparency. Correlation scale is between -2 and 2 10−4. For the coordinates for A, B and C views see the legend of Fig 2. Neurological convention (Right = Right). (PDF) [file pone.0194422.s001.pdf]

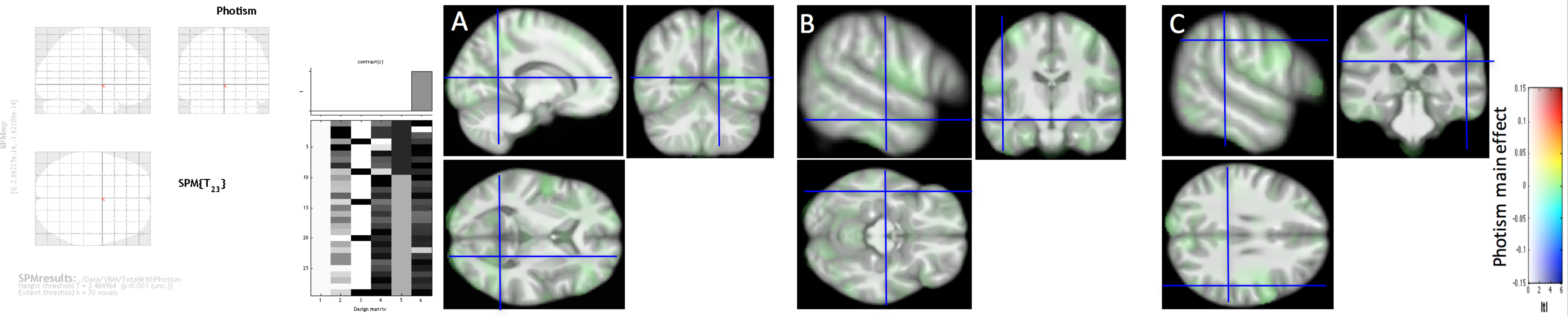
Photism
SPMmip
[0, 2.84217e-14, -1.42109e-14]
SPM{T23}
contrast(s)
Design matrix
SPMresults: ./Data/VBM/TotalWithPhotism
Height threshold T = 3.484964 {p<0.001 (unc.)}
Extent threshold k = 70 voxels
A
B
C
Photism main effect
0.15
0.1
0.05
0
-0.05
-0.1
-0.15
0 2 4 6
|t|
