# Supplementary figures and images for: Magnetic resonance imaging does not reveal structural alterations in the brain of grapheme-color synesthetes
Source: PLoS One. 2018 Apr 4;13(4):e0194422. doi: 10.1371/journal.pone.0194422 (PMC5884511; doi:10.1371/journal.pone.0194422)

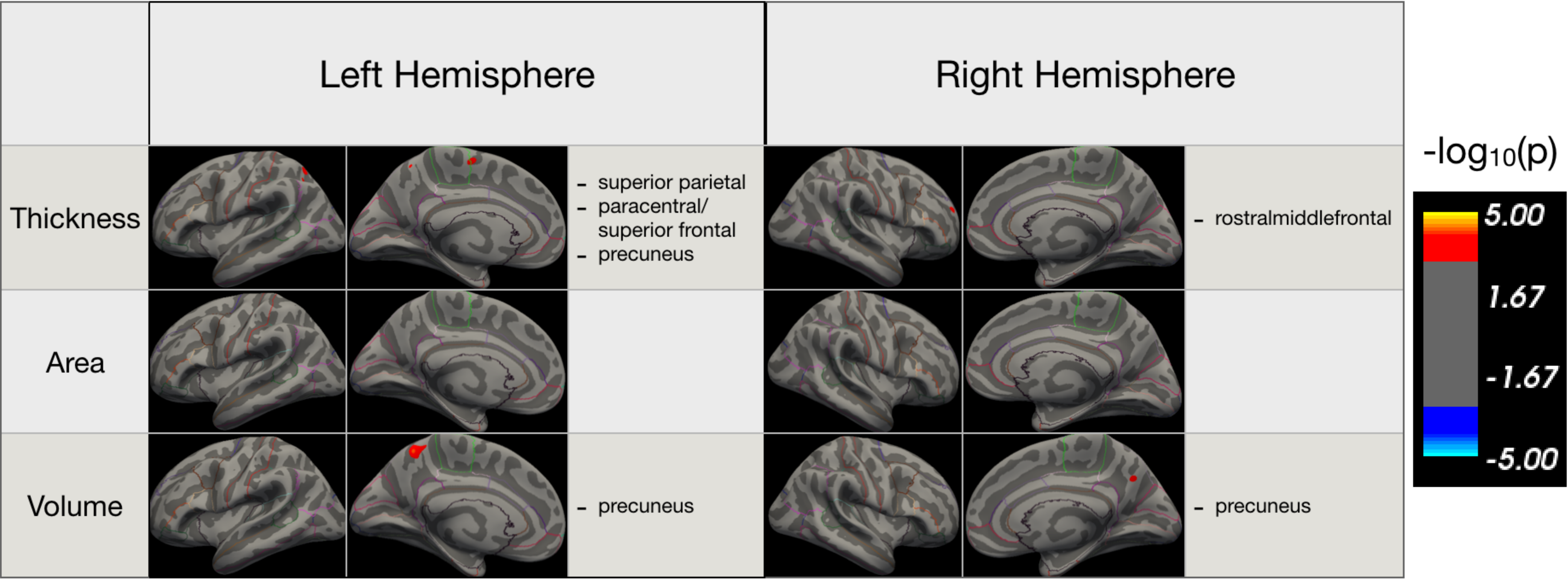

Supplement: S2 Fig — Differences in 48 controls vs. 32 synesthetes for cortical thickness (mm), area (mm2) and volume (mm3) for left and right hemispheres (Cluster-forming threshold p<0.001). For each parameter, regions where differences were detected are indicated. No region survived the FWEc at the cluster level. (PDF) [file pone.0194422.s002.pdf]
