# Supplementary material for: Magnetic resonance imaging does not reveal structural alterations in the brain of grapheme-color synesthetes
Source: PLoS One. 2018 Apr 4;13(4):e0194422. doi: 10.1371/journal.pone.0194422 (PMC5884511; doi:10.1371/journal.pone.0194422)
Supplement: S1 Text — Material and Methods. (PDF) [file pone.0194422.s003.pdf]

Material and Methods

Surface based analysis.

For each subject, cortical thickness of the cortical ribbon was computed on a uniform grid (comprised of vertices) with 1 mm spacing across both cortical hemispheres, with the thickness being defined by the shortest distance between the grey–white and pial surface models. Thickness measures were mapped to the inflated surface of each participant's brain reconstruction, allowing visualization of data across the entire cortical surface (i.e. gyri and sulci) without being obscured by cortical folding. Each subject's reconstructed brain was then morphed to an average spherical surface representation that optimally aligned sulcal and gyral features across subjects. This procedure provides accurate matching of morphologically homologous cortical locations among participants on the basis of each individual's anatomy while minimizing metric distortions. This transform was used to map the thickness measurements into a common spherical coordinate system. Data were resampled for all subjects into a common spherical coordinate system. The data were then smoothed on the surface tessellation using an iterative nearest-neighbor averaging procedure (Gaussian smoothing kernel along the cortical surface with a full-width-at-half-maximum of ~13 mm).
We compared the maps of cortical volume, thickness and surface of controls and synesthetes by performing a vertex-wise univariate analysis using the general linear model (GLM) as implemented in Freesurfer, independently for left and right hemispheres. As for the volume analysis we added brain volume of each hemisphere, sex and age as cofactors. No cluster was found at $p<0.01$ uncorrected for multiple comparison, for the contrast Synesthetes>Controls. We used this threshold trying to reproduce the results reported in [12]. However, differences were found ($p<0.001$) for Controls vs Synesthetes (see S2-Fig).
