# Supplementary material for: Magnetic resonance imaging does not reveal structural alterations in the brain of grapheme-color synesthetes
Source: PLoS One. 2018 Apr 4;13(4):e0194422. doi: 10.1371/journal.pone.0194422 (PMC5884511; doi:10.1371/journal.pone.0194422)
Supplement: S1 Table — Average response time (inverse of the mean inverse RT) in synesthetic Stroop tasks: CC: Congruent stimuli in Color task; IC: Incongruent Color; CP: Congruent Photism task; IP: Incongruent Photism. “Synesthesia Strength” (also called photism strength) = (IC-CC)–(CP-CC). (see for details in [48]). (PDF) [file pone.0194422.s004.pdf]

Subject's Name	*Study*	*Age (years)*	*Sex*	*Synesthesia Strength*	*CC*	*IC*	*CP*	*IP*	*CP-CC*	*IC-CC*
syn02a	1	26	1	--	--	--	--	--	--	--
syn03a	1	36	2	-107	465	453	560	593	95	-12
syn04a	1	28	2	553	621	1115	562	618	-59	494
syn05a	1	28	2	100	583	627	527	615	-57	43
syn07a	1	56	1	29	685	725	696	802	12	40
syn08a	1	52	2	-77	403	432	509	623	106	30
syn09a	1	24	2	-75	560	570	645	848	85	11
syn10a	1	31	2	20	573	624	604	655	31	51
syn11a	1	33	2	-107	652	676	783	980	131	24
syn12a	1	50	1	-10	444	487	497	617	53	43
syn01b	2	21	2	-107	443	454	561	685	119	12
syn02b	2	21	2	52	466	543	491	543	25	77
syn05b	2	30	2	20	452	501	480	568	29	49
syn07b	2	26	2	-60	573	612	672	828	99	39
syn08b	2	35	1	69	484	585	516	619	32	101
syn10b	2	31	2	40	556	633	593	689	37	77
syn11b	2	28	2	146	643	782	636	681	-7	138
syn12b	2	33	2	136	597	683	548	646	-50	86
syn13b	2	29	2	30	668	744	714	795	46	76
syn20b	2	23	2	19	506	556	537	637	31	50
syn22b	2	28	1	--	--	--	--	--	--	--
syn26b	2	27	1	-19	497	528	547	690	50	31
syn27b	2	24	2	-4	547	582	586	676	39	36
syn30b	2	27	2	416	466	844	427	449	-39	378
syn31b	2	24	2	20	525	573	553	639	27	47
syn32b	2	33	2	-57	456	477	534	620	78	21
syn33b	2	30	2	-30	447	457	487	525	40	10
syn39b	2	26	2	48	440	523	475	582	35	83
syn40b	2	42	2	--	--	--	--	--	--	--
syn41b	2	23	2	-15	484	514	529	678	45	30
syn45b	2	27	2	10	539	564	554	691	16	25
syn48b	2	22	1	-73	487	529	602	770	115	42
