# Supplementary material for: Magnetic resonance imaging does not reveal structural alterations in the brain of grapheme-color synesthetes
Source: PLoS One. 2018 Apr 4;13(4):e0194422. doi: 10.1371/journal.pone.0194422 (PMC5884511; doi:10.1371/journal.pone.0194422)
Supplement: S2 Table — * subject not used in sulci and surface-based morphometry analyzes. (PDF) [file pone.0194422.s005.pdf]

Subject's Name	*Study*	*Age*	*Sex*	*Subject's Name*	*Study*	*Age*	*Sex*
con1a	1	49	2	con03b	2	26	1
con2a	1	24	1	con04b	2	26	1
con3a	1	31	2	con06b	2	23	2
con4a	1	21	1	con14b	2	37	2
con5a	1	31	2	con15b	2	28	2
con6a	1	32	1	con16b	2	24	1
con7a	1	33	2	con17b	2	27	2
con8a	1	28	1	con18b	2	27	2
con9a	1	24	2	con19b	2	33	1
con10a	1	24	1	con21b	2	26	2
con11a	1	23	2	con23b	2	25	2
con12a	1	24	1	con24b*	2	24	2
con13a	1	24	2	con25b	2	24	2
con14a	1	25	1	con28b	2	29	2
con15a	1	24	2	con29b	2	25	2
con16a	1	25	1	con34b	2	24	2
con17a	1	25	1	con35b	2	30	2
con18a	1	23	1	con36b	2	27	2
con19a	1	25	1	con37b	2	34	2
con20a	1	59	2	con38b	2	26	2
con195a	1	26	2	con42b*	2	32	2
con284a	1	38	1	con43b	2	38	1
con292a	1	35	1	con44b	2	37	2
con293a	1	40	1	con46b	2	27	1
con06a	1	36	2	con47b	2	26	2
