# Supplementary material for: Magnetic resonance imaging does not reveal structural alterations in the brain of grapheme-color synesthetes
Source: PLoS One. 2018 Apr 4;13(4):e0194422. doi: 10.1371/journal.pone.0194422 (PMC5884511; doi:10.1371/journal.pone.0194422)
Supplement: S3 Table — (PDF) [file pone.0194422.s006.pdf]

		Study 1	*Study2*	*Study1 and Study2*
Sample	Synesthetes	10	22	32
	Controls	25	25	50
Age mean ± sd (range)	Synesthetes	36.4 ± 11.82 (24 - 56)	27.73 ± 5.06 (21 - 42)	30.44 ± 8.64 (21 - 56)
	Controls	29.8 ± 8.95 (21 - 59)	28.2 ± 4.40 (23 - 38)	29 ± 7.04 (21 - 59)
	p-value (T-test)	0.13	0.74	0.43
